# Zebrafish: A Versatile and Powerful Model for Biomedical Research

**DOI:** 10.1002/bies.70080

**Published:** 2025-10-18

**Authors:** Sundus Siddiqui, Hiba Siddiqui, Emna Riguene, Michail Nomikos

**Affiliations:** ^1^ College of Medicine, QU Health Qatar University Doha Qatar

**Keywords:** animal model, biomedical research, human disease, model organism, zebrafish

## Abstract

Zebrafish (*Danio rerio*) have become a versatile model in precision medicine, bridging fundamental biology with translational applications. Their optical transparency, rapid development, and high genetic conservation with humans enable real‐time imaging and cost‐efficient high‐throughput screening. Advances in CRISPR/Cas9, prime editing, and morpholino approaches have expanded their utility for modeling diverse human diseases. In addition to well‐established roles in cardiovascular, neurological, metabolic, oncological, and infectious disease research, emerging applications include non‐invasive larval urine assays, functional validation of rare human variants, host–microbiome interactions, and automated behavioral profiling for neuropsychiatric conditions. Limitations such as species‐specific lipid metabolism and limited antibody availability remain, yet recent integration of single‐cell transcriptomics, computational modeling, and machine learning is enhancing translational relevance. Collectively, these innovations position zebrafish as a scalable and powerful platform for disease modeling and personalized therapeutic strategies, underscoring their growing impact in the evolving landscape of precision medicine.

## Graphical abstract

Zebrafish are vital for advancing precision medicine, offering a genetically tractable and optically transparent vertebrate model with strong genetic homology to humans. They enable rapid and cost‐effective disease modeling and drug discovery, accelerating insights into cardiovascular, neurological, metabolic, and oncological disorders through gene‐editing technologies and high‐throughput phenotypic analysis.

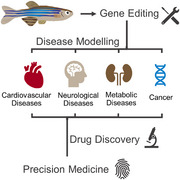

## 1 Introduction

Human biology is inherently complex, making direct experimentation and observation both challenging and, in many cases, ethically unfeasible. To overcome these challenges, scientists use model organisms‐species that are extensively studied due to a thorough understanding of their genetics, physiology, and development. These organisms serve as simplified systems to investigate complex biological processes in a more manageable and ethically acceptable way. Model organisms play a crucial role in biomedical research by simplifying the study of fundamental mechanisms such as development, gene function, and disease progression [1]. They also provide ethical and practical advantages, allowing experiments that cannot be performed in humans, and they accelerate drug discovery and toxicological screening, helping translate findings more quickly to human health. The selection of these models is based on several key factors, such as genetic tractability, short generation time, cost‐efficiency, and ease of maintenance and breeding in laboratory conditions. The most commonly used models in laboratories are bacterium *Escherichia coli*, baker's yeast *(Saccharomyces cerevisiae)*, fruit fly *(Drosophila melanogaster)*, roundworm *(Caenorhabditis elegans)*, African clawed frog *(Xenopus laevis)*, zebrafish (*Danio rerio*), and mouse (*Mus musculus*), which were historically used as standard models, each with specific advantages tailored to specific biological questions [2].

Among these, the zebrafish (*Danio rerio*) has rapidly gained recognition as a valuable vertebrate model for biomedical research. Its increasing use in modeling human disease arises from a combination of biological, practical, and genetic features, making it particularly suitable for in vivo studies. Indeed, zebrafish present a high similarity of genomic and physiological conservation with humans, approximately 70% of human genes have at least one zebrafish ortholog, and 84% of genes known to be linked with human diseases have zebrafish counterparts. The 70% genetic similarity between zebrafish and humans originates from comparative genomic studies that estimate the proportion of protein‐coding genes in humans with at least one zebrafish ortholog. However, this number primarily reflects gene presence as genetic similarity does not always imply complete functional conservation. Many genes may have diverged in regulatory elements, expression patterns, or biochemical functions. The 30% genetic divergence includes genes unique to humans, zebrafish‐specific paralogs resulting from teleost whole‐genome duplication, and genes with significantly altered functions, which limits direct translational relevance in some contexts [3, 4]. This level of genetic identity enables the modeling of a wide range of genetic and complex diseases, ranging from developmental disorders to cancer and cardiovascular diseases.

One of the most distinctive advantages of the zebrafish model is the optical transparency of its embryos and larvae, allowing a real‐time imaging of cellular dynamics and organ development. This feature is particularly important in the context of developmental biology and high‐resolution microscopy, making the scientists able to observe dynamic process in real time in live organisms [5]. Moreover, in addition to the external fertilization and rapid embryogenic development, the major organs systems are formed within 24–72 h postfertilization, making zebrafish a great time‐efficient system to investigate the vertebrate embryology [6].

Furthermore, zebrafish are also highly valuable for genetic manipulation. Several techniques are increasingly used, such as morpholino antisense oligonucleotides, CRISPR/CAS 9 genome editing, and transgenic approaches, to understand the gene function or to replicate human diseases mutations [7]. Due to their prolific breading, zebrafish is ideal for subsequent genetic approaches and high throughput phenotypic assays to conduct a large‐scale mutagenesis screening. All these features are particularly crucial in bioassay development, where reproductivity, quantifiability, and scalability are important. In terms of physiology, zebrafish models are becoming increasingly used as models for cardiovascular conditions, neurological, metabolic, and infectious diseases [8]. Interestingly, zebrafish are anatomically and functionally similar to human organs such as heart, blood vessels, nervous system, kidney, and liver. Notably, zebrafish have proven particularly useful in modeling cardiomyopathies, arrhythmias, and vascular defects, providing a platform for rapid screening of therapeutic compounds in a vertebrate system [9].

Moreover, zebrafish husbandry is more cost‐effective than mammalian models. Their small size and compatibility with multi‐well plate formats allow for automated imaging and behavioral tracking systems, which facilitate large scale chemical and genetic screening assays [10]. These advantages combine to position zebrafish as a unique bridge between in vitro cell culture system and more complex mammalian models [11].

In spite some constraints, with a different lipid metabolism, difficult histological sectioning due to their reduced dimensions, and the variability introduced by aquatic husbandry systems, ongoing technological innovations are helping to overcome these obstacles. As bioassays become more sophisticated, the zebrafish model evolves, offering a strong scalable, and translationally relevant platform for modeling biomedical research [9].

In this review, we provide an update overview of the zebrafish model, emphasizing its key benefits, current applications in biomedical research, and future prospects in diseases modeling and therapeutic development. This review provides a novel perspective by integrating recent genome editing tools—such as CRISPR, prime editing, and morpholinos—within the zebrafish model, highlighting their role in advancing precision disease modeling. Unlike previous reviews, it identifies emerging yet underexplored areas, including larval urine analysis and targeting splicing/nonsense mutations. It also offers a comparative view of zebrafish versus traditional models, addressing a gap in balanced evaluations and outlining future directions in functional genomics. Disease areas were included based on recent advancements in zebrafish‐based genetic modeling, translational relevance, and representation of both communicable and non‐communicable conditions. Rather than employing a systematic review framework, this article offers a critical and integrative overview of key studies published over the last 20 years, with special focus on recent contributions that highlight the zebrafish's versatility in biomedical research field.

## 2 Zebrafish as a Model System

### 2.1 Evolutionary and Genetic Similarity to Humans

Among these, a particularly influential factor is the high degree of genomic similarity and conserved homology between zebrafish and other vertebrate species, including humans, which underpins their relevance for translational research. The zebrafish genome encodes at least 25 000 genes, and more than 70% of human genes have been identified to contain zebrafish orthologs [12]. Augmenting the substantial level of gene conservation between humans and zebrafish is the fact that approximately 80% of pathological human genes known to cause disease to have a clear ortholog identified in zebrafish (Table 1) [13, 14].

**TABLE 1:** A comparative overview of zebrafish, mice, and humans highlights key similarities and differences relevant to biomedical research.

Features	Zebrafish	Mice	Humans
Genetic similarity to humans (%)	∼70% of human genes have at least one zebrafish ortholog ^[^ ^3^ ^]^	∼ 85% genetic similarity to humans ^[^ ^11^ ^]^	100%
Transparency for imaging	High (especially in larvae, and transparent strains like Casper) ^[^ ^12^ ^]^	Low, imaging typically requires invasive methods	N/A
High‐throughput drug screening	Very high, larvae can be screened in multi‐well plates ^[^ ^13^ ^]^	Moderate, limited by size, cost and time	Low, ethical and logistical limitations
Disease modeling efficiency	High for many diseases, especially developmental, cardiovascular, and cancer models	High, many complex diseases can be modelled ^[^ ^14^ ^]^	Direct, but experimental manipulation is not feasible
Ethical & cost considerations	Low cost, fewer ethical limitations compared to mammals ^[^ ^8^ ^]^	Higher cost, stricter ethical regulations	Very high ethical concerns and costs

Zebrafish models effectively overcome the limitations of using other animals to model human diseases, such as mice. For instance, the costs of utilizing rodents are higher as compared to zebrafish. To further illustrate this limitation, mice are a lot larger than zebrafish, meaning that they have greater housing requirements. Moreover, the genes of mice are harder to manipulate. Additionally, mice offspring develop in the uterus; hence, limiting external visibility of fetal development [15].

Historically, common animal models, like chickens and rabbits, have played crucial roles in studying human diseases and developing new treatments. However, their high cost, space requirements and ethical considerations regarding animal welfare, make the use of these mammalian models more limited. Therefore, zebrafish offer a compelling alternative due to its easy maintenance and small size, and reduced housing cost compared to larger vertebrates. Moreover, the ethical concerns regarding animal suffering are alleviated by the use of zebrafish, as they are less sentient than mammals. This approach follows the 3Rs principles in animal research, advocating for the use of models with reduced cognitive capacity and lower distress levels [16].

Zebrafish provide a genetically comparable, cost‐effective and ethically great alternative to traditional mammalian models, that make them an increasingly valuable toll in human disease research.

### 2.2 Technical Advantages in Zebrafish Research

Zebrafish offer unique advantages for studying human diseases and exploring potential therapies. Key features include external fertilization, rapid development, high fecundity, ease of genetic manipulation, and suitability for real‐time imaging and chemical screening. Tools like CRISPR/Cas9, prime editing and morpholino oligonucleotides (MOs) have expanded the use of this in vitro animal model as a bridge between cell‐based assays and whole animal studies, especially for low cost, high throughput applications [17].

#### 2.2.1 External Fertilization and Embryo Transparency

Zebrafish embryos develop externally and are transparent, allowing real‐time, non‐invasive imaging of developmental and cellular processes [5]. Combined with high embryo output, this enables large‐scale, microscopy‐based phenotypic screens. Thousands of live embryos can be imaged within days, making zebrafish a powerful model for drug screening and disease modeling [18].

#### 2.2.2 Rapid Development of Zebrafish

Major zebrafish organs are formed within 24–48 h after fertilization, facilitating quick studies across developmental stages (from early embryogenesis to organogenesis) [19]. This supports high‐throughput screening (HTS) of 1000 to 10 000 assays per day. Large‐scale replicates increase statistical power and help detect rare responses, often using genetically diverse wild‐type strains. Zebrafish models are also increasingly used to assess drug safety in humans [17].

#### 2.2.3 High Fecundity of Zebrafish: A Key Advantage for High‐Throughput Biomedical Research

Zebrafish reach sexual maturity in 3–4 months and can produce hundreds of embryos weekly [20, 21]. Their high fecundity and low maintenance costs make them ideal for large‐scale pharmacological and genetic studies. These traits support efficient HTS and mutagenesis screening, surpassing many higher vertebrate models [10].

#### 2.2.4 Ease of Genetic Manipulation (CRISPR, Morpholinos, Transgenics)

Zebrafish are highly amenable to genetic engineering. Tools like prime editing, CRISPR/Cas9, and MOs allow precise genome editing to model human diseases. Prime editing enables precise nucleotide modifications, insertions, and deletions without causing double‐stranded DNA breaks, making it ideal for the simulation of somatic and germline mutations in order to mimic human genetic diseases. The remarkable efficacy of prime editing in zebrafish embryos, combined with its ability to model diseases and assess therapeutic strategies, demonstrates its high potential for translational applications in biomedical research [22].

CRISPR/Cas9 enables precise knock‐in and knock‐out mutations in zebrafish, facilitating studies of genes associated with diseases like atrial fibrillation and neurological disorders [23, 24, 25]. MOs provides a cost‐effective tool to suppress gene activity in large‐scale research [26, 27]. Tissue regeneration enhancer elements (TREEs), non‐coding DNA sequences, enhance understanding of tissue regeneration, offering promise for regenerative medicine [28, 29].

Zebrafish also support studies of RNA editing and post‐transcriptional changes, with enzymes like ADAR playing critical roles in neural and immune function [30]. Collectively, these tools enable modeling of human diseases, testing therapies, and advancing fundamental biology, reinforcing zebrafish as a vital model in genetics (Table 2).

**TABLE 2:** Genetic tools and their applications in zebrafish research.

Technique	Purpose/use case	Strengths	Limitations	Representative studies
Prime editing	Precise nucleotide changes, insertions, deletions	No double‐stranded breaks	Still developing in zebrafish, variable efficiency	[25]
CRISPR/Cas 9	Studying gene function through gene knockout/knock in, disease modeling	Rapid and straightforward knockout generation, germline transmission	Cutting efficiency, HDR efficiency, mosaicism and off target effects	[26, 27]
Morpholinos (MOs)	Gene silencing: Translation blocking, splicing interference	Widely used and established, versatility, rapid and efficient	Off‐target effects, transient knockdown, stringent controls required	[28]
MOs transgenic/TREEs	Stable transgenic tool to block translation and prevent splicing of pre‐mRNA	Revolutionized gene manipulation, cost‐effective and relatively easy to use	Off‐target effects, injection variability, transient effect	[29]
RNA editing studies (ADAR)	Posttranscriptional studies	Unique genomic context, tissue specificity	Limited mechanistic understanding and functional redundancy	[30]

#### 2.2.5 Ability to Conduct Live Imaging and Chemical Screening

Zebrafish's small size and transparency make them ideal for live imaging using fluorescent markers to observe cells and organs dynamically [31]. They are widely used in automated chemical screening platforms to assess drug effects at cellular and organism levels. Behavioral analysis further support studies of psychoactive compounds and cancer therapies. Advanced hardware/software now supports high‐throughput handling and imaging of large zebrafish populations [32].

Zebrafish combine rapid development, high fecundity, ease of genetic modifications, and real‐time imaging capabilities, making them an efficient and powerful tool for high‐throughput modeling of human diseases and drug screening.

## 3 Applications in Human Disease

**FIGURE 1:**
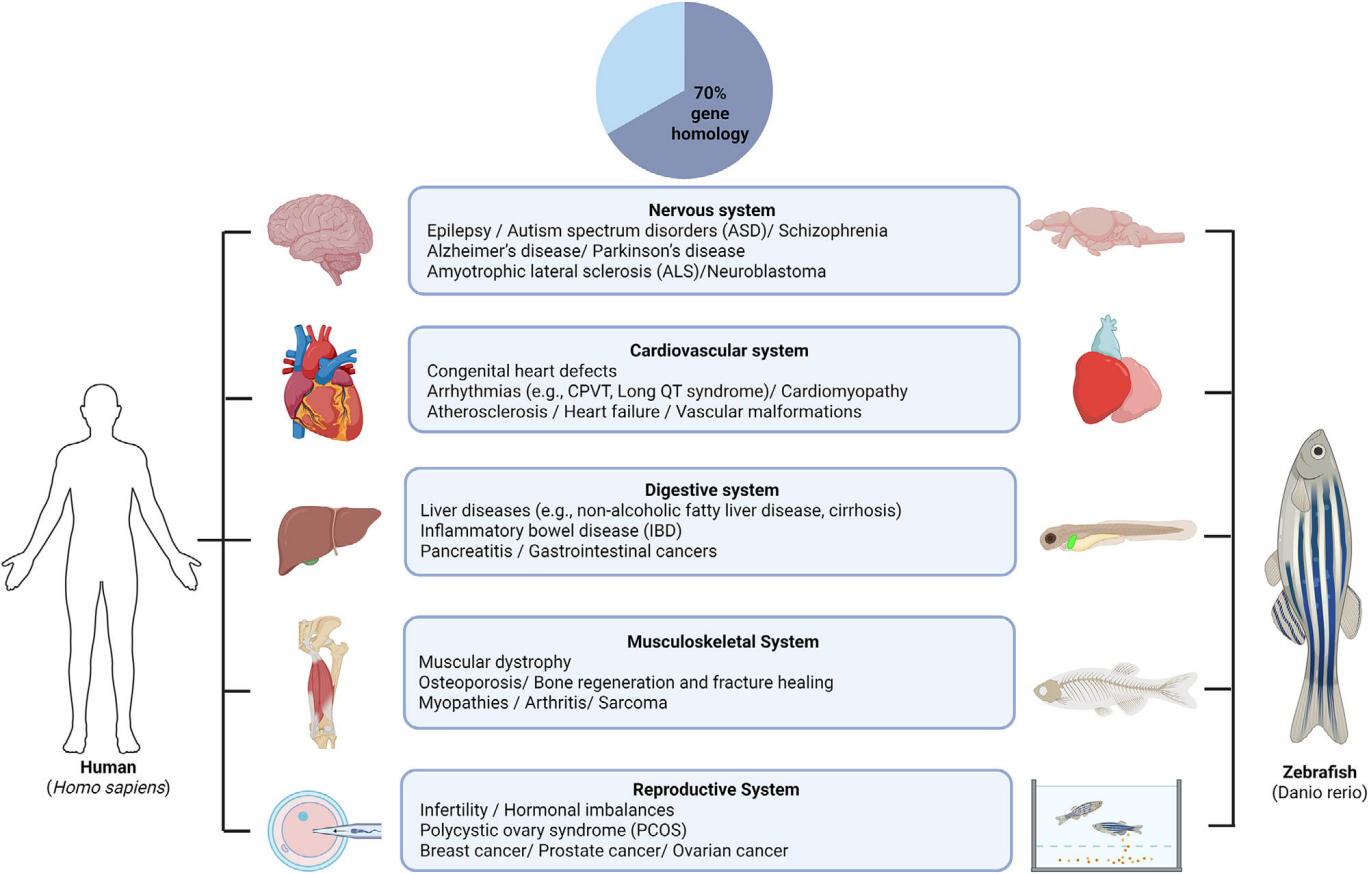
Illustration of the comparison of human organs and zebrafish model to study various diseases. Each human organ system corresponds to zebrafish anatomical structures, as well as nervous, cardiovascular, digestive, musculoskeletal, and reproductive systems. Zebrafish‐specific organ structures are highlighted as tools for investigating developmental biology and pathophysiological of human diseases. Created in BioRender. Nomikos, M. (2025) https://BioRender.com/pvt5p5w.

The zebrafish model has become an invaluable tool in biomedical research due to its genetic and physiological similarities to humans. These features enable efficient modeling of genetic mutations, diseases mechanisms, and therapeutic strategies (Figure 1). Zebrafish have been utilized to investigate pathological pathways of neurodegenerative diseases (NDD) and other neural disorders [33, 34] Studies using transgenic, morpholinos, or chemical treatments have also explored developmental and metabolic disorders and congenital abnormalities [35, 36, 37, 38]. The versatility of such model organism for studying pathological mechanisms of different diseases has been well documented [15, 39]. Table 3 summarizing these studies provide key details on models, treatment strategies, and main findings that highlight the importance of zebrafish model in diseases research.

**TABLE 3:** Representative zebrafish studies in biomedical research. Summary of key zebrafish models used in neurodegenerative, developmental, and metabolic disorder research, detailing experimental models, treatment methods, duration, and main findings.

	Author and year	Model	Dose/treatment	Duration of treatment	Main findings
Neurodegenerative disease research	Vijayanathan et al., 2017	Adult zebrafish, microinjection of neurotoxin	6.25–100 mg/kg into ventral diencephalon	Single injection	Ablation of >85% dopaminergic neurons; used to study neurodegeneration and regeneration ^[^ ^34^ ^]^
	Wasel et al., 2020	Zebrafish with targeted gene edits or morphants	Variable genetic modification	Embryonic–larval stages	Applied to model mechanistic Parkinson's disease pathways ^[^ ^35^ ^]^
Developmental research	Harish et al., 2023	Morpholino or mutation affecting fgf8a	Genetic knockdown/mutation	Early embryogenesis	Disrupted neural patterning, mesoderm and organogenesis via FGF signaling pathways ^[^ ^36^ ^]^
	Reynaud et al., 2008	Morpholino targeting lox genes	Morpholino injection	Embryogenesis	Undulated notochord, truncated AP axis, tail bending, reduced head size^[^ ^37^ ^]^
Metabolic disorder research	Li et al., 2023	HFD and overfeeding obesity models	HFD and DIO	9 weeks	Showed increased lipid accumulation, hepatic steatosis; transcriptome alterations^[^ ^38^ ^]^
	Carnovali et al., 2018	High‐fat diet in adult zebrafish	HFD	Weeks	Altered glucose/insulin levels, influenced bone metabolism^[^ ^39^ ^]^

### 3.1 Cardiovascular Diseases

**FIGURE 2:**
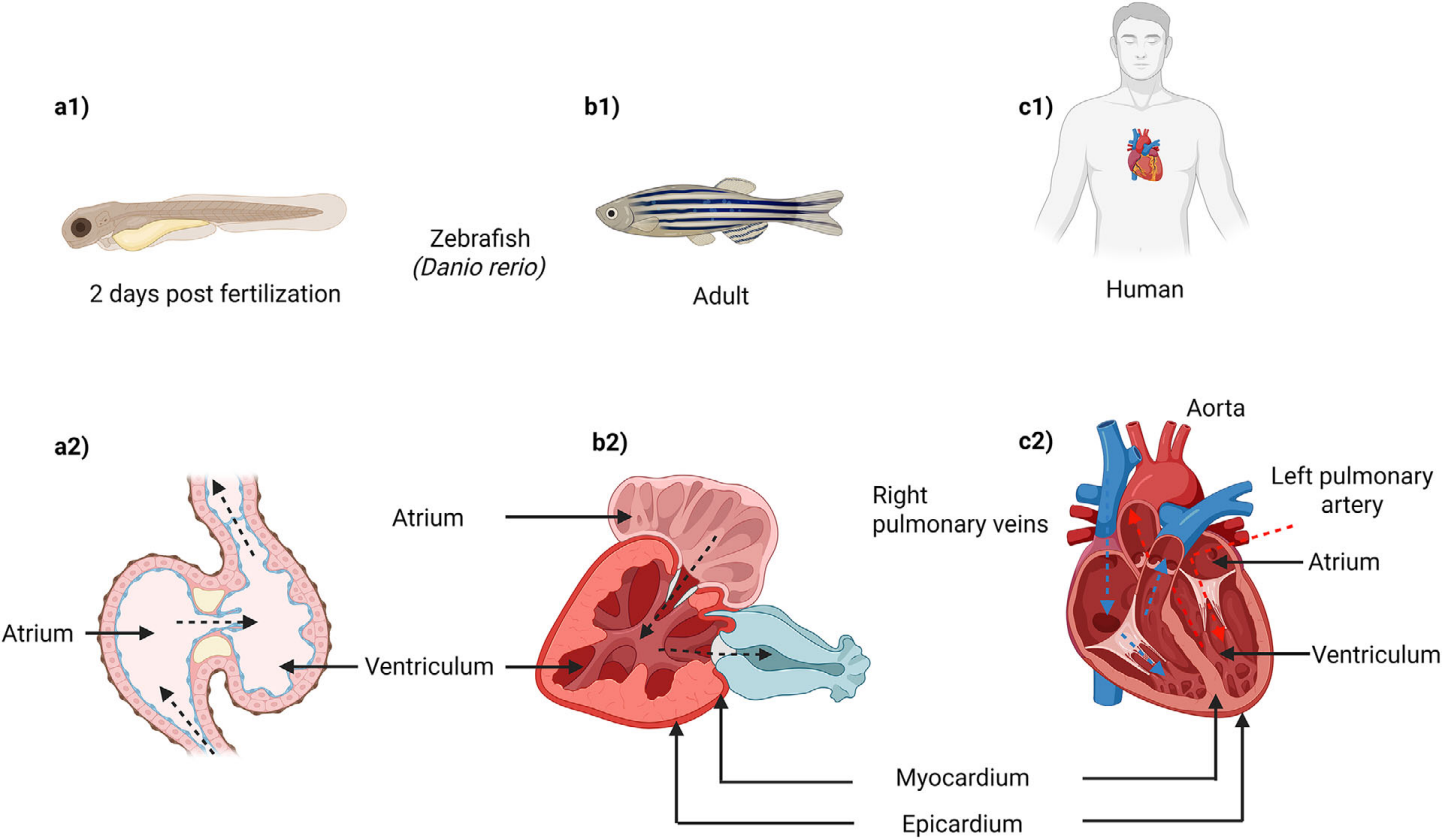
Comparative overview of cardiovascular systems in zebrafish and human. This figure illustrates the similarities and differences between zebrafish and human cardiovascular systems. Panels (a1) and (b1) shows the zebrafish embryo at 2 days post fertilization and in adulthood, respectively, with a two chambered heart visible in both stages. Panel (c1) depicts a human with the heart's location indicated. Panels (a2) and (b2) illustrate the internal structure of the zebrafish embryonic and adult heart, each comprising a single atrium and ventricle supporting single circulation. Panel (c2) displays the human heart with distinct left and right atria and ventricles, aorta, pulmonary arteries, and veins, allowing dual circulation. Labels indicate structural components such as the myocardium and epicardium. Blood flow was showed in arrows. Created in BioRender. Nomikos, M. (2025) https://BioRender.com/9vsytli.

Zebrafish are widely used to study cardiovascular diseases due to the conserved cardiac architecture and cellular composition. Despite lacking lungs and pulmonary circulation, their two‐chambered heart (one atrium and one ventricle), maintains systemic blood flow similar to mammals. The bulbus arteriosus, a specialized, non‐contractile chamber connected to the aorta, functions as a pressure capacitor sustain circulation through the gills and body. These features, combined with transparent embryos and genetic accessibility, make zebrafish ideal for cardiovascular research (Figure 2).

#### 3.1.1 Modeling Congenital Heart Defects

Zebrafish represents an effective model for heart defects. For example, mutations in calmodulin (*CALM*) gene linked to arrhythmias, such as long QT syndrome, have been studied in zebrafish embryos. Injection of mutant calmodulin cRNAs revealed morphological changes and arrhythmic heartbeats at 72 h post‐fertilization, mimicking human phenotypes [40] model is also instrumental in modeling hypertrophic cardiomyopathy (HCM). Knockout and missense mutations in the zebrafish *mybpc3* gene (ortholog of human *MYBPC3*) lead to thickened ventricles, cardiac edema and reduced heart rate, mimicking the effects seen in human patients with HCM. Additionally, novel splice‐site mutations in *MYBPC3* further cause reduced cardiomyocyte counts, and impaired cardiac output, highlighting zebrafish as a robust system for understanding the pathophysiology of arrhythmias and cardiomyopathies and for testing therapies [41, 42, 43].

#### 3.1.2 Drug Testing for Heart Failure and Vascular Diseases

In addition to its role in genetic studies, zebrafish are well‐suited for cardiovascular drug testing, due to their unique features. For instance, extracts from *Cynodon dactylon* and *Sida acuta* have been shown to modulate cardiac function in zebrafish embryos. *Cynodon dactylon* increase heart rate and blood flow velocity, while *S. acuta* reduces significantly both parameters, underscoring their opposite cardiotropic effects [44]. Zebrafish are also used in HTS for evaluating efficacity and cardiotoxicity of novel compounds. The ability to perform live imaging of the heart allows researchers to study the dynamics of heart failure and the impact of drugs on cardiac performance at the cellular level, facilitating the development of new therapeutic strategies.

#### 3.1.3 Studying Blood Viscosity and Vascular Remodeling

Manipulating blood viscosity in zebrafish, such as through *gata1* knockdown, has allowed studies on hemodynamic and vascular remodeling. Reduced viscosity lowers wall shear stress, affecting angiogenesis and vessel development, particularly in cardiac and caudal vein plexus regions. This provides insights into vascular diseases and potential treatments [45].

#### 3.1.4 Studying Cardiac Action Potentials and Conduction Pathologies

Zebrafish cardiac action potential closely resembles those in humans, with a comparable plateau phase and ion flux. This similarity supports their use in studying conditions disorders such as sick sinus syndrome, long QT syndrome, and atrial fibrillation [46].

### 3.2 Neurological Disorders

#### 3.2.1 Modeling Neurodevelopmental Disorders

Zebrafish have proven highly valuable for studying neurodevelopmental disorders such as Rett syndrome, autism spectrum disorder (ASD), and epilepsy. Their transparent embryos, rapid development, and genetic tractability make them suitable for investigation brain development and its related pathologies. Notably, *shank3* gene mutations, lined to ASD, have been modeled in zebrafish, resulting in ASD‐like social behavior deficits and altered neural connectivity, aiding therapeutic discovery [47]. Similarly, Rett syndrome, caused by *MECP2* mutations, has been studied using zebrafish to understand synaptic plasticity disruption and motor alteration, offering a platform to probe molecular mechanisms [48]. Zebrafish have also been used to model epilepsy, exhibiting seizures in response to genetic mutations or pharmacological agents, thereby supporting antiepileptic drugs discovery and seizure mechanism studies.

#### 3.2.2 Sleep and Wakefulness Physiology

Zebrafish have proven to be a great model for studying sleep physiology and wakefulness due to their genetic and neurochemical similarities to humans. They present sleep‐like behaviors, maintain circadian rhythms, and regulate neurotransmitters in similar ways to mammals, which make them ideal for investigating the neural basis of sleep disorders. Indeed, zebrafish are able to respond to pharmacological compounds, notably, melatonin, that induce sleep‐like states, and have conserved orexin/hypocretin pathways regulating arousal. These findings support high‐throughput experimental techniques. Together, these features make zebrafish an excellent model for examining both fundamental mechanisms and potential therapeutic strategies for such neurological disorders [49].

#### 3.2.3 Neurodegenerative Diseases

The global rise in NDD, especially in aging populations, underscores the need for effective models. Characterized by progressive neuronal loss and motor/cognitive decline, NDD remain poorly treated due to their clinical heterogeneity and complex pathology. Zebrafish offer key benefits, high fecundity, transparency, and genetic similarity to humans, making them powerful models for diseases such as Alzheimer's disease, Parkinson's disease (PD), and amyotrophic lateral sclerosis (ALS), yielding important insights into disease mechanisms and potential therapeutic approaches [50, 51, 52].

#### 3.2.4 Parkinson's Disease

Zebrafish have been used to model PD such as mitochondrial dysfunction and oxidative stress [53]. Exposure to neurotoxins like 6‐hydroxydopamine (6‐OHDA) induces dopaminergic neuron loss, motor deficit, and dopamine reduction in zebrafish, mimicking human PD pathology [54]. Zebrafish also model α‐synuclein (α‐syn) accumulation, a hallmark of PD. Transgenic lines expressing human α‐syn mimic PD‐related neuronal damage, which can be mitigated by CLR01, a molecular tweezer that reduces toxic protein aggregates [55]. Importantly, zebrafish with *Pink1* deletions display dopaminergic neuron loss, an outcome not achieved in mouse models with *Parkin*, *Pink1*, and *DJ‐1* knockouts, showing the superior feasibility of zebrafish in modeling human PD [56].

#### 3.2.5 Alzheimer's Disease

Transgenic zebrafish expressing human amyloid precursor protein (APP) replicate amyloid beta accumulation and neurodegeneration observed in AD [57]. These models allow a real‐time imaging of plaque formation and neuronal loss, while also supporting studies on neuroinflammation and drug testing, providing a comprehensive platform for AD research [51].

#### 3.2.6 Amyotrophic Lateral Sclerosis (ALS)

ALS, a progressive neurodegenerative disease affecting motor neurons, and characterized by the presence of protein inclusions in the affected neurons. Transgenic zebrafish expressing mutant *SOD1*, implicated in ∼20% of familial ALS cases, replicate motor deficits and spinal motor neurons degeneration [58]. Their transparent embryos facilitate live imaging of neuronal activity, providing a powerful tool to assess ALS progression and screen novel therapies [59].

### 3.3 Cancer Research

Zebrafish emerged as a valuable model for studying cancer biology and metastasis due to the strong conservation of tumor progression pathways with humans. Many human oncogenes, tumor suppressors, and cell cycle regulators have orthologs in zebrafish. However, some cancer‐related genes, such as breast cancer 1 early onset (BRCA1) and leukemia inhibitory factor (LIF), are not conserved [15].

#### 3.3.1 Advantages of Xenograft Models in Zebrafish

The optical transparency of zebrafish embryos and pigment‐deficient strains like Casper fish enable real‐time visualization of tumor growth and metastasis.

Genetic manipulation techniques, such as knockdown, knockout, overexpression and transgenesis, make zebrafish ideal for modeling specific cancers and studying tumor‐host interactions. Additionally, transgenic lines with fluorescent vasculature or organs further improve in vivo imaging of tumor progression [60].

#### 3.3.2 Use in Studying Leukemia, Melanoma, and Breast Cancer

Zebrafish are widely used to study various cancer types. Notably, expression of human oncogenes such as *AKT1* in neural cells via Tol2‐mediated integration models early tumorigenesis, including inflammation and immune cell recruitment. In this context, preneoplastic cells attract macrophages through the Sdf1b‐Cxcr4b signaling pathway, promoting oncogenic proliferation. In adult zebrafish, oncogenes can be introduced through transgene electroporation into somatic cells, successfully modeling tumor initiation and progression in immune competent systems [61]. Zebrafish have also been instrumental in modeling metastasis stages in human cancers. Cutaneous melanoma models, for instance, form tumors near epithelial surfaces of adult zebrafish, aligning with human melanoma [62]. Real‐time imaging techniques, such as confocal microscopy, provide deep insight into tumor dynamics and support the development of targeted therapies [63].

#### 3.3.3 High‐Throughput Drug Screening for Personalized Medicine

Zebrafish cancer models support forward and reverse genetic screens to identify cancer‐linked genes and pathways. Gene‐editing tools including MO, CRISPR, TALENs, and EFNs, enable precise genetic modifications. These models are also well suitable for high throughput drug screening, facilitating rapid testing of therapeutic compounds an in advancing targeted medicine strategies [64, 65].

### 3.4 Infectious Diseases

Transgenic zebrafish lines expressing fluorescent markers in immune cells, such as neutrophils, macrophages, and T cells, have become essential tools for studying host‐pathogen interactions, cell autonomous immunity and inflammation, in bacterial infections like *Salmonella*, *Shigella*, *Pseudomonas*, and *Streptococcus* [66, 67]. *Shigella* serotypes, for example, establish persistent infections in zebrafish, where bacteria adopt a pleomorphic shape within macrophages, impairing rod morphology as a survival strategy [68]. Zebrafish larvae's transparency allows real‐time visualization of these interactions and inflammatory responses relevant to human disease. Zebrafish can be naturally infected through the gastrointestinal tract, gills, damaged skin, or bloodsucking parasites. To investigate gut microbiota interactions more precisely, gnotobiotic zebrafish model have been developed.

#### 3.4.1 Modeling Tuberculosis, Viral Infections, and Sepsis in Zebrafish

Viral infections can also be modeled using zebrafish. For example, Herpes simplex virus (HSV‐1) infection in adult zebrafish showed viral entry in the brain via the 3‐O sulfated heparan receptor, homologous to its human counterpart. This triggers the expression of type I interferon and related genes in larvae. In tuberculosis research, tumor necrosis factor (TNF)was shown to activate the same mitochondrial ROS (mROS) pathway in both human and zebrafish macrophages, leading to ROS production via reverse electron transport. Hence, zebrafish serves as a platform for evaluating mROS pathway inhibiting drugs [69].

#### 3.4.2 Zebrafish as a Model for Human Innate Immunity

The zebrafish innate immune system closely mirrors human responses. In a *Streptococcus agalactiae* (GBS) model, dose‐dependent larval death and brain penetration by the ST‐17 strain were observed, implicating the GBS capsule and toxin production in virulence. Elevated IL‐1β and IL‐6 cytokine levels confirmed proinflammatory responses similar to human infections. Such findings support zebrafish as a model for studying bacterial pathogenesis and guiding vaccine development [70].

### 3.5 Metabolic Disorders

#### 3.5.1 Diabetes and Lipid Metabolism Disorders

Zebrafish are valuable for modeling both type I and type 2 diabetes. In type 1 diabetes mellitus models, selective β‐cells destruction by streptozotocin injection led to vascular complications such as microangiopathy and ischemia mimicking human pathology [71]. Diabetes type 2 was simulated through *PDX1* gene knockdown, resulting in reduced β‐cell numbers and elevated glucose levels. These models effectively replicate microvascular complications due to hyperglycemia. Moreover, treatments with metformin or glimepiride in overfed zebrafish lowered blood glucose, reflecting their clinical efficacy. Overall, zebrafish offer reliable tools to model diabetic pathology and treatment responses [72].

#### 3.5.2 Phenylketonuria and Lysosomal Storage Disorders in Zebrafish

Zebrafish are increasingly used to study rare metabolic conditions such as lysosomal storage diseases (LSDs), affecting approximately 1 in 5000 newborns and often lead to neurodegeneration. Zebrafish share conserved metabolic pathways and organ systems with humans. For example, their early kidney (pronephros), models cellular uptake defects seen in LSDs [73]. In phenylketonuria (PKU), zebrafish mutants generated via CRISPR/Cas9 with *PAH* gene defects showed toxic phenylalanine accumulation, neurobehavioral deficits, and developmental abnormalities similar to human PKU. These models also revealed disruptions in signaling pathways linked to LSD, with some pharmacological interventions successfully reversing disease phenotypes [74]. The optical transparency of zebrafish embryos, combined to the high‐throughput capacity and enhanced genetic manipulability make the zebrafish as an ideal in vivo model for investigating human diseases mechanism and discovering new treatments.

## 4 Limitations and Challenges of Zebrafish as a Model

### 4.1 Differences From Mammalian Physiology

Despite their many advantages, zebrafish also have notable physiological differences from mammals, which can limit their prevalence in certain disease models. For example, while zebrafish share similar pancreatic structure with humans, their genetic differences affect modeling of metabolic diseases like obesity. Zebrafish leptin protein shares only 19% identity with the human protein and lacks expression in adipocytes [75]. Consequently, zebrafish with leptin receptor deficiency exhibit dysregulated glucose homeostasis, rather than obesity, hyperphagia, and hyperlipidemia seen in mammals’ models like mice. Therefore, mice remain more appropriate for studying lipid metabolism and related disorders [76]. This discrepancy illustrates a broader issue. Even zebrafish share similar biological pathways as humans, they may fail to fully replicate complex physiological responses that depend on mammalian‐ specific structures, pathways, or tissue organization. Consequently, scientists need to carefully consider whether the results from zebrafish studies can be applicable directly to humans, or they need to be confirmed in a mammalian model [77, 78].

### 4.2 Challenges in Disease Modeling

One of the most significant constraints is the anatomical simplicity of zebrafish relative to mammals. The zebrafish cardiovascular system, for example, comprises a two‐chambered heart with a single circulatory loop, lacking a distinct pulmonary circuit. This limits its utility in modeling complex human cardiovascular conditions such as myocardial infarction, atherosclerosis, and pulmonary hypertension [79]. Additionally, zebrafish respire through gills rather than lungs, making them fundamentally unsuitable for studying human respiratory diseases such as chronic obstructive pulmonary disease (COPD), asthma, or cystic fibrosis in their full complexity [80].

The immune system also presents notable differences. While zebrafish possess both innate and adaptive immunity, their adaptive immune system does not reach full maturity until approximately 4–6 weeks after fertilization. This temporal limitation complicates modeling of diseases reliant on mature T‐ and B‐cell responses, such as autoimmune disorders or chronic viral infections, particularly in larval or juvenile stages [67]. Zebrafish can sometimes be useful in effective modeling of immune‐ related disease. However, this has led to a debate among scientists, trying to balance the potential using zebrafish as a research model and the need for careful interpretation of immunological findings [81, 82].

Moreover, zebrafish are ectothermic and maintained at temperatures around 28°C, which diverges from mammalian thermoregulation. Differences in metabolic rate, enzyme kinetics, and temperature‐sensitive gene expression may lead to species‐specific drug pharmacokinetics, thereby reducing the predictive value of certain pharmacological studies [83]. While zebrafish enable speed and scalability for drug design, the translation of pharmacokinetics and toxicity data to human remains inconsistent, with various compounds that are efficient on zebrafish but fail in mammalian preclinical models [84, 85].

The use of antisense morpholino oligonucleotide for gene knockdown in zebrafish is effective only during early development, partly due to p53‐induced neural apoptosis. In addition, zebrafish lack certain central nervous system structures, notably corticospinal and rubrospinal tracts, limiting their relevance in modeling upper motor neuron disorders [86]. Moreover, genome editing tools, like CRIPSR/Cas9, start to overcome some of such genetic limitations, such as off‐target effects, that remains challenging and complicate the interpretation of disease phenotypes [87].

Practical limitations also exist. The small size of zebrafish embryos complicates the histological sectioning, and a shortage of validated antibodies impedes detailed histological and immunological analysis [88]. Due to these technical limitations, the confirmation of molecular and cellular results becomes challenging, making it even more important to verify the results in mammalian systems [89].

### 4.3 Need for Standardization in Zebrafish Research

Variations in diet and husbandry practices can significantly affect zebrafish health and study outcomes. For example, spontaneous neoplasms have been observed in some laboratory colonies but not in others, suggesting potential effects of environmental factors or carcinogens in the diet on the tumor development [90]. Furthermore, while human tumor xenografts can be studied in zebrafish, immune rejection remains a challenge. Solutions like irradiating or genetic immunosuppression of zebrafish can mitigate this issue but may affect the tumor environment, limiting the translational relevance of such models.

The current state of reproducibility in zebrafish research remains variable, largely due to a lack of universal standards for husbandry, genetic background, and experimental protocols [91]. These inconsistencies can lead to divergent phenotypic outcomes across laboratories. We now emphasize the need for coordinated efforts to develop and adopt standardized guidelines, such as those proposed by the Zebrafish Husbandry Association (ZHA) and other international consortia, to improve inter‐laboratory reliability and translational validity. Without careful standardization of used approaches, discrepancies in terms of reliability of zebrafish models will arise, as separate laboratories need to reach contradictory outcomes when investigating the same biological problem. Acknowledging and critically addressing these shortcomings is crucial to better define the circumstances where zebrafish serve as a robust model, and those where mammalian or other systems are more appropriate [92].

Reproducibility remains a significant challenge in zebrafish research, as outcomes can vary depending on strain differences, environmental factors (such as diet, light cycles, or tank density), and technical protocols for genetic manipulation or imaging. Even subtle variations in laboratory practices can lead to conflicting results when addressing similar biological questions. To improve reliability, international initiatives such as the Zebrafish Information Network (ZFIN) and community guidelines for husbandry and experimental design are promoting greater standardization and transparency [92, 93]. Recent discussions also emphasize the need for preregistration of experimental protocols and wider adoption of FAIR (Findable, Accessible, Interoperable, Reusable) data practices to enhance reproducibility and cross‐laboratory validation [94]. Addressing these issues is critical to ensuring that zebrafish remain a robust and reliable model in translational biomedical research.

## 5 Zebrafish as a Robust Research Model

**FIGURE 3:**
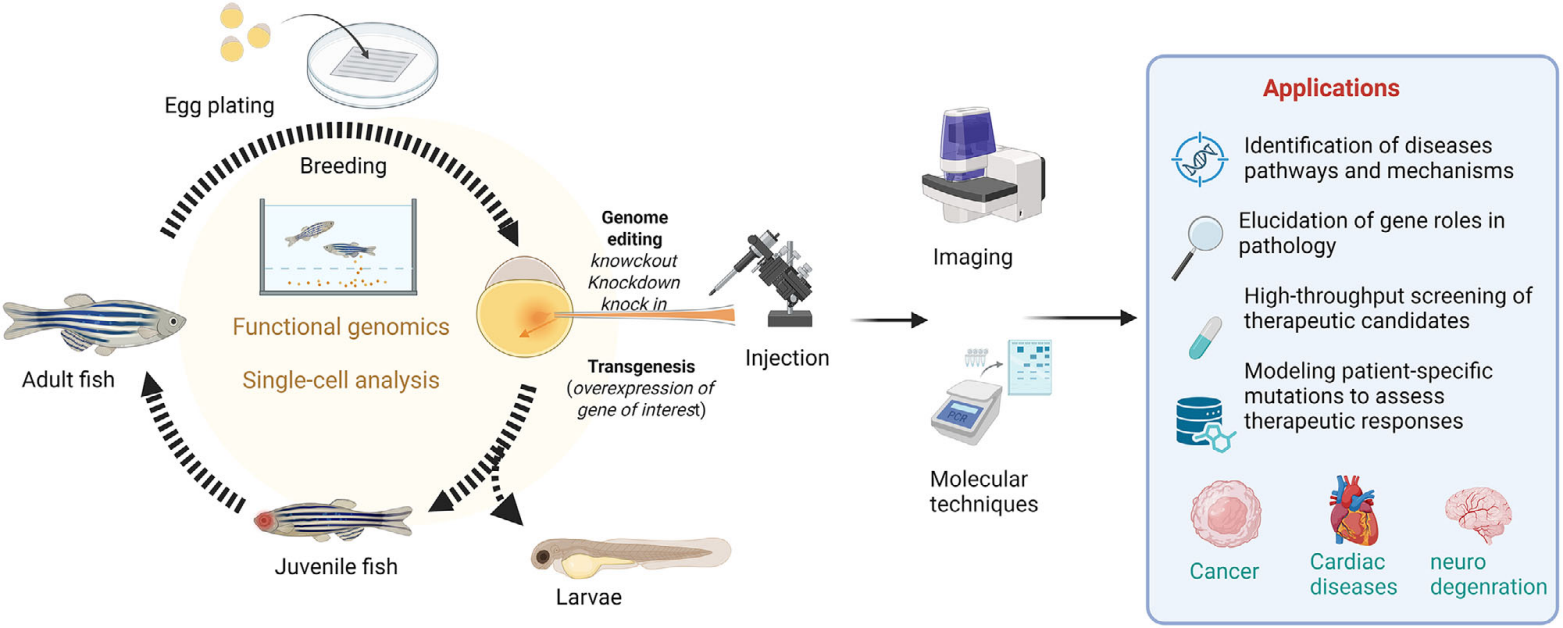
Workflow of zebrafish applications in disease modeling and drug discovery. Illustration of the workflow for using zebrafish as a model in diseases research and drug discovery. Zebrafish are genetically modified to study gene function, beginning with egg breeding and plating and progressing through knockout, knockdown, and transgenesis. Following microinjection and development into larvae and adults, data is collected using imaging and molecular techniques. These approaches have a wide range of applications, including diseases pathway identification, gene function analysis, high throughput drug screening and modeling patient‐specific mutations for therapeutic evaluation in cancer, cardiac diseases, and neurodegeneration. Created in BioRender. Nomikos, M. (2025) https://BioRender.com/318ftrb.

Zebrafish have emerged as a versatile and powerful model organism with high potential for advancing biomedical research. Their genetic tractability, rapid development, and transparent embryos make them well‐suited for studying human diseases and investigating intricate biological pathways. Researchers continue exploring innovative strategies to broaden their use in diseases modeling and therapeutic discoveries (Figure 3).

Despite their increased utility, challenges highlight the urgent need for deep interpretation of zebrafish‐based data and underscore the importance of integrating zebrafish studies with mammalian.

### 5.1 Genome Annotation and Functional Studies

The annotated zebrafish genome, with high similarity to the human genome, offers a strong foundation for investigating gene functions and disease mechanisms. Advanced techniques, such as RNA sequencing (RNA‐Seq) and single‐cell sequencing allow researchers to uncover unannotated genes and regulatory domains, helping to map genotype‐phenotype relationships more precisely [95].

### 5.2 Advancements in Gene Editing & Disease Modeling

Zebrafish have significantly contributed to gene‐editing progress, especially through CRISPR/Cas 9, which enable targeted mutation that mimic patient‐specific genetic changes [96]. This had made zebrafish a valuable model for studying polygenic and complex diseases influenced by both genetics and environment, such as cardiovascular, neurological and metabolic disorders. Their genetic diversity further enhances the relevance of disease susceptibility research [97].

### 5.3 Expanding Pharmacological and Translational Research

The optical transparency of zebrafish embryos allows real‐time visualization of drug adsorption, distribution, and clearance. This feature, along with their rapid development and cost‐effectiveness, enables efficient screening of drug candidates. While translation to human patients remains a challenge, zebrafish models have shown promising results in predicting drugs efficacity and potential side effects that patients can have [98].

### 5.4 Integration With Other Model Systems

Zebrafish research gains further impact when integrated with mammalian models, offering complementary strengths in drug screening and pharmacological testing. Combining zebrafish data with computational modeling and artificial intelligence helps to predict diseases outcomes and supports the development of personalized therapies, increasing translational relevance [99, 100]. Overall, these advances underscore the zebrafish's growing role in bridging basic research and clinical applications, strengthening its position as a powerful tool in functional genomics, disease modeling, and translational medicine.

### 5.5 Ethical Considerations

Zebrafish are often portrayed as an ethically favorable alternative to mammalian models due to their small size and external development, yet ethical scrutiny remains crucial. Zebrafish are considered protected vertebrates from the point of independent feeding (∼5 days postfertilization), prompting requirements for humane treatment beyond this stage [101]. Recent evidence supports the presence of nociceptive pathways in early larvae, arguing for the use of anesthesia and analgesia in potentially painful procedures across developmental stages. The 2023 Federation of European Laboratory Animal Science Associations (FELASA) working group emphasizes that fish, including zebrafish, should receive pain relief and refinement in housing and procedures, with anesthesia protocols and analgesic immersion treatments recommended [102]. On euthanasia, zebrafish physiology complicates common methods like concussion or electrical stunning. As a result, hypothermic shock is gaining acceptance under guidance from European Union and American Veterinary Medical Association frameworks [103]. Collectively, these evolving standards underscore that zebrafish, despite perceived ethical advantages, require rigorous welfare measures, including proper anesthesia, analgesia, humane euthanasia, and environmental enrichment in compliance with the 3Rs (Replacement, Reduction, Refinement) principle. By following these frameworks, researchers ensure ethical compliance while leveraging the experimental advantages of zebrafish.

## 6 Future Directions

Zebrafish continue to be a highly versatile model for studying human diseases due to their optical transparency, rapid development, genetic tractability, and cost‐effective maintenance. Looking ahead, advances in genome editing, including CRISPR/Cas9 refinements and prime editing, promise more precise modeling of human genetic variants. Emerging technologies such as single‐cell RNA sequencing, high‐resolution imaging, and AI‐driven behavioral and image analysis will enhance our understanding of cellular processes and disease mechanisms [104]. Innovative experimental approaches, including microfluidic systems for non‐invasive physiological monitoring and gnotobiotic zebrafish for studying host–microbiome interactions, are expanding the model's utility. Zebrafish are increasingly used to validate rare human genetic variants, support standardized modeling of neurodevelopmental and psychiatric disorders, and assess environmental toxicology through transgenic biosensors and high‐throughput screening [105]. Furthermore, integrating zebrafish data with mammalian and human datasets via cross‐species bioinformatics and machine learning is expected to improve translational relevance and predictive capacity, bridging the gap between preclinical research and clinical application [106].

Collectively, these developments indicate that zebrafish will remain at the forefront of experimental and translational research, enabling novel insights into disease mechanisms, therapeutic strategies, and personalized medicine. Analysis of aging and cellular senescence, including processes such as telomere shortening and neurodegeneration, is also becoming feasible in adult zebrafish, providing new opportunities for gerontology research [107].

## 7 Conclusions

Zebrafish is a robust and adaptable system for studying human diseases, offering a distinct advantage in visualization, genetic manipulation, and the ability of large‐scale investigations. Ongoing advancements in molecular tools, imaging methods, and the integration of computational approaches promise to increase the importance of zebrafish in biomedical research. Studies in zebrafish have directly informed clinical research; for example, models used in NDD have oriented the identification of neuroprotective compounds, while metabolic disorders models shed light onto pathways relevant to human therapies. These discoveries enable deeper understanding of complex diseases processes and support the development of tailored treatments, firmly establishing zebrafish as a critical bridge for translating basic science into clinical applications.

## Author Contributions

All authors approved the submitted manuscript and contributed equally to the study.

## Conflicts of Interest

The authors declare no conflicts of interest.

## Data Availability

Data sharing not applicable to this article as no datasets were generated or analyzed during the current study.
